# Carbimazole-associated Pancreatitis: Report From Western India

**DOI:** 10.1210/jcemcr/luad155

**Published:** 2023-12-26

**Authors:** Vyankatesh Shivane, Nihar Mehta, Ajay Jhaveri, Saba Samad Memon

**Affiliations:** Department of Endocrinology, Jaslok Hospital and Research Centre, Mumbai 400026, Maharashtra, India; Department of Cardiology, Jaslok Hospital and Research Centre, Mumbai 400026, Maharashtra, India; Department of Gastroenterology, Jaslok Hospital and Research Centre, Mumbai 400026, Maharashtra, India; Department of Endocrinology, Seth GS Medical College and KEM Hospital, Mumbai 400012, Maharashtra, India

**Keywords:** carbimazole, Graves disease, pancreatitis

## Abstract

Pancreatitis is a very rare complication of methimazole and carbimazole therapy. We describe a case of possible carbimazole-associated pancreatitis. A 41-year-old Asian man (with no comorbidities) reported to the hospital with atrial fibrillation and a fast ventricular rate. He was diagnosed with hyperthyroidism due to Graves disease. His rhythm was reverted with amiodarone, and carbimazole was initiated at 15 mg daily for the medical management of Graves disease. Fifteen days later, he presented with acute severe abdominal pain and vomiting with elevated serum amylase 387 U/L (reference range, 28-100 U/L) and lipase levels 206 U/L (reference range, 13-60 U/L). Magnetic resonance imaging showed a bulky pancreas with extensive extrapancreatic fat stranding suggestive of acute pancreatitis. Considering the possibility of carbimazole-related pancreatitis, the drug was withheld. He was managed conservatively, and his pancreatic enzymes normalized within 1 week. The observation suggests that the pancreatitis was a consequence of the therapy with carbimazole. Although it is a rare occurrence, patients taking carbimazole who report abdominal discomfort and vomiting should be evaluated for pancreatitis.

## Introduction

The drugs used in the medical management of hyperthyroidism are methimazole, carbimazole, and propylthiouracil. Carbimazole is a prodrug that gets converted to an active metabolite of methimazole. It has a lesser propensity for hepatotoxicity than propylthiouracil. The rare side effects with its use are agranulocytosis and cholestatic jaundice. Its association with drug-induced pancreatitis is rarer. Only 10 cases of thionamide-induced pancreatitis have been described in a literature review (PubMed, Embase), 8 with methimazole and 2 with carbimazole (1‐3). We describe a case of possible carbimazole-associated pancreatitis.

## Case Presentation

**Figure 1.**
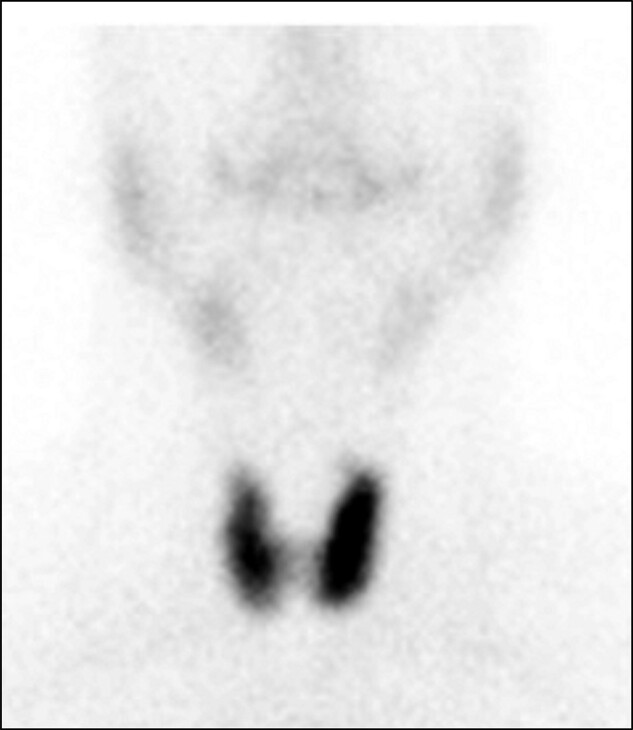
^99m^Tc scan showing diffuse uptake in the entire thyroid gland—overall uptake 3.4%, right lobe uptake 1.5%, and left lobe 2% (normal uptake is 0.4%-1.7%).

A 41-year-old Asian man with no comorbidities (no history of alcohol abuse disorder, nonsmoker) reported to the hospital with sudden-onset epigastric pain and 3 episodes of vomiting. On examination, he had tachycardia (heart rate 180/min) with an irregular pulse. He had normal blood pressure (120/70 mm Hg) and an unremarkable systemic examination. Electrocardiogram suggested atrial fibrillation with a fast ventricular rate, whereas a 2-dimensional echocardiogram was normal. He was treated with amiodarone 150 mg bolus followed by infusion for 24 hours, with which his tachyarrhythmia resolved. An ultrasound of the abdomen was unremarkable except for grade 1 fatty liver. Pancreatic enzymes were normal (amylase 61 U/L [reference range, 28-100 U/L] and lipase 20 U/L [reference range, 13-60 U/L]). On workup for atrial fibrillation, he was found to be thyrotoxic—undetectable thyrotropin (TSH) less than 0.005 µLU/mL (reference range, 0.4-4.0 µLU/mL) with high free thyroxine (T4) 1.91 ng/dL (reference range 0.8-1.8 ng/dL [24.5 pmol/L; range, 10.3-23.2 pmol/L]) and high normal free triiodothyronine (T3) 3.7 pg/mL (reference range, 2.8-4 pg/mL [5.7 pmol/L; range, 4.3-6.1 pmol/L]). He had elevated levels of antithyroid peroxidase and antithyroglobulin antibody with diffuse uniform uptake on a ^99m^ Tc thyroid scan (Fig. 1). TSH receptor antibody was not tested. He was discharged on carbimazole 15 mg per day along with metoprolol 25 mg twice daily, amiodarone 200 mg once daily, apixaban 2.5 mg once daily, and rosuvastatin 10 mg once daily.

**Figure 2.**
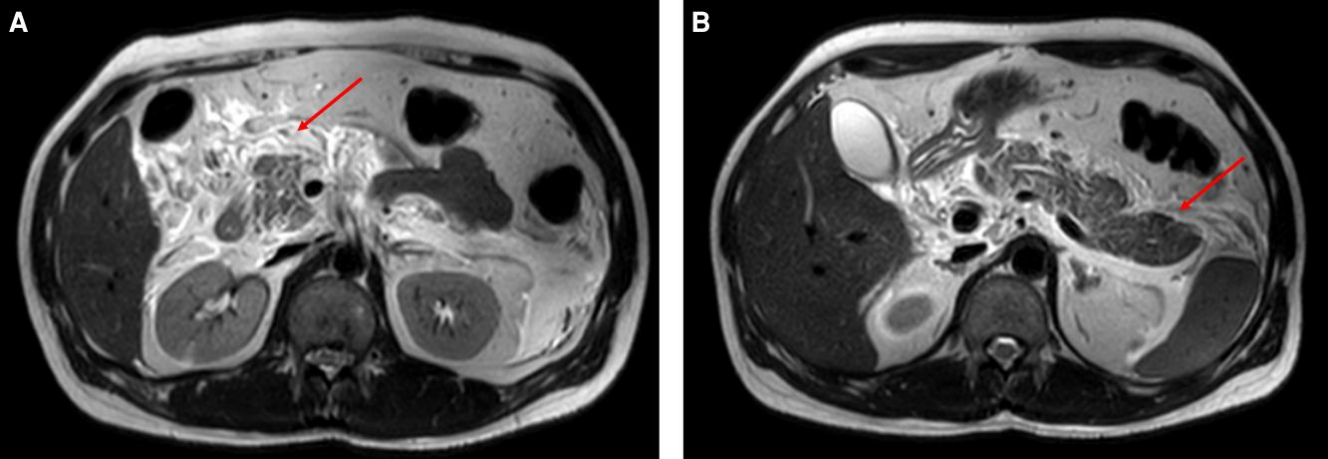
Magnetic resonance cholangiopancreatography images showing bulky pancreas with peripancreatic fat stranding—A, head of pancreas and B, body and tail arrows indicate areas of fat stranding.

He returned to the emergency department 15 days after his initial visit with severe acute abdominal pain and vomiting. He had elevated serum amylase 387 U/L (reference range, 28-100 U/L), lipase levels 206 U/L (reference range, 13-60 U/L), and a bulky pancreas on ultrasound. His biochemical investigations are summarized in Table 1. Magnetic resonance cholangiopancreatography (MRCP) and magnetic resonance imaging of the abdomen depicted a bulky pancreas with extensive extrapancreatic fat stranding and fluid, which were suggestive of acute pancreatitis (Fig. 2). There was no dilation of the gallbladder, common bile duct, pancreatic duct, or intrahepatic biliary radicles. The magnetic resonance imaging was not suggestive of autoimmune pancreatitis. He did not have hypercalcemia (serum calcium 8.2 mg/dL [reference range, 8.6-10 mg/dL], 2.04 mmol/L [reference range, 2.15-2.49 mmol/L]) or hypertriglyceridemia (triglycerides 108 mg/dL [reference range <150 mg/dL], 1.22 mmol/L [reference range <1.69 mmol/L]). There was no prior history of pancreatitis or other autoimmune disorders. The severity of pancreatitis was graded as mild.

**Table 1.** Temporal trends in biochemical investigations of the patient

	Normal range	Day 1	Day 3	Day 15	Day 16	Day 21	Day 120
Amylase	28-100 U/L	61 U/L		**387 U/L**		**105 U/L**	
Lipase	13-60 U/L	20 U/L		**206.6 U/L**		47.2 U/L	
Hemoglobin	13-17 g/dl	15.4 g/dl				13.5 g/dl	
WBC	4000-10000/µl	10590/µl				8400/µl	
Neutrophil percentage	55-70%					64.2%	
SGOT	10-40 U/L	27 U/L					
SGPT	10-41 U/L	32 U/L					
Alkaline phosphatase	40-129 U/L				80.5 U/L		
Bilirubin	0.2-1.3 mg/dl (3.42-22.4 µmol/l)	0.27 mg/dl (4.62 µmol/l)					
Free T3	2.8-4 pg/ml (4.3-6.1 pmol/l)		3.7 pg/ml (5.7 pmol/l)	2.66 pg/ml (4.09 pmol/l)			**0.56 pg/ml (0.86 pmol/l)**
Free T4	0.8-1.8 ng/dl (10.3-23.2 pmol/l)		**1.91 ng/dl (24.5 pmol/l)**	1.43 ng/dl (18.4 pmol/l)			**0.18 ng/dl (2.37 pmol/l)**
TSH	0.4-4.0 µlU/ml		**< 0.005 µlU/ml**	**0.018 µlU/ml**			**>100 µlU/ml**
Triglycerides	<150 mg/dl (<1.69 mmol/l)	122 mg/dl (1.37 mmol/l)			108 mg/dl (1.22 mmol/l)		
Total cholesterol	<200 mg/dl (<5.17 mmol/l)				148 mg/dl (3.83 mmol/l)		
LDL cholesterol	60-130 mg/dl (1.55-3.36 mmol/l)	165 mg/dl (4.27 mmol/l)			96.1 mg/dl (2.48 mmol/l)		
VLDL cholesterol	<30 mg/dl (<0.78 mmol/l)				21.7 mg/dl (0.56 mmol/l)		
HDL cholesterol	>40 mg/dl (>1.03 mmol/l)	33 mg/dl (0.85 mmol/l)			30.3 mg/dl (0.78 mmol/l)		
Calcium	8.6-10 mg/dl (2.15-2.49 mmol/l)	9.2 mg/dl (2.29 mmol/l)			8.2 mg/dl (2.04 mmol/l)		
Phosphorus	2.5-4.5 mg/dl (0.8-1.45 mmol/l)				3.7 mg/dl (1.19 mmol/l)		
Albumin	3.9 – 4.9 gm/dl				3.9 gm/dl		

SI units are mentioned in brackets wherever conventional units are also added. Abnormal values are marked in bold

HDL – high-density lipoprotein, LDL - low-density lipoprotein, SGOT – serum glutamic-oxalacetic transaminase, SGPT – serum glutamic-pyruvic transaminase, T3 - triiodothyronine T4 – thyroxine, TSH – thyroid stimulating hormone, VLDL – very low-density lipoprotein, WBC – white blood cell

## Treatment

Considering the possibility of carbimazole-related pancreatitis, the drug was withheld. Pancreatitis was managed conservatively. The patient gradually improved over the next few days, and the pancreatic enzymes normalized on day 21 since the first presentation (6 days from pancreatitis onset).

## Outcome and Follow-up

To further manage hyperthyroidism, the patient underwent radioiodine ablation (12 millicurie) and is currently hypothyroid on replacement and doing well as of the last follow-up (4 months). His human leukocyte antigen (HLA) typing showed A*02:xx A*26:xx, B*08:xx B*40:xx, DRB1*03:xx DRB1*14:xx, and DRB3*.

## Discussion

This report describes a case of Graves disease with possible carbimazole-related acute pancreatitis from Western India. The diagnosis of Graves disease was based on a radionuclide scan. Chronic use of amiodarone can lead to a false-negative interpretation of the technetium thyroid scan due to its high iodine content. In our patient, amiodarone exposure was present for 2 days prior, and the presence of thyroid uptake with suppressed salivary gland uptake despite amiodarone exposure was suggestive of hyperfunctioning thyroid. Hyperthyroidism in a patient on amiodarone could be due to the effect of amiodarone per se. However, in the present patient, the thyroid dysfunction is unlikely due to amiodarone, as the onset of amiodarone-related thyroid dysfunction usually occurs after few months of therapy (4). Preponderant T3 synthesis and elevated T3/T4 ratio are typically observed in Graves disease due to high expression of D2 in Graves thyroid tissue. However, it was not seen in our patient. This could have been due to the effect of amiodarone (which the patient received before testing) affecting the conversion of T4 to T3. The patient fulfilled the criteria for drug-induced pancreatitis as 1) there was a temporal association between the onset of acute pancreatitis to carbimazole administration, 2) the absence of other causes of pancreatitis (alcohol, gall stones, hypercalcemia, hypertriglyceridemia), 3) reversal of elevated pancreatic enzymes after withdrawal of carbimazole, and 4) normal MRCP. The patient was not rechallenged due to the possibility of recurrence, and rechallenge criteria could not be considered. Although the criteria include normal endoscopic retrograde cholangiopancreatography, MRCP was performed in our patient, which was normal. Further, there were no MRCP features of primary autoimmune pancreatitis, like diffuse thickening with peripancreatic halo and loss of lobulation. Additionally, the patient did not have any prior history of pancreatitis. The patient was on other drugs at the onset of pancreatitis: amiodarone, metoprolol, apixaban, and rosuvastatin. The role of metoprolol and apixaban in causing pancreatitis is less likely as his pancreatic enzymes resolved despite the continuation of metoprolol to date. Further, evidence of rosuvastatin-induced pancreatitis is less robust as it belongs to class IV based on probability classification by Badalov et al (5). In contrast, amiodarone belongs to class Ib and was also stopped during the admission with acute pancreatitis, and its role in causality cannot be excluded entirely (6).

The existing case reports on thionamide-induced pancreatitis are presented in Table 2. The age at presentation in our patient is similar to the age group (range, 18-80 years) described in the literature. Most patients described are female (9/10), which contrasts with the male sex on our patient. The reason for female sex predilection is unclear; it could be due to, per se, more common occurrence of thyroid dysfunction and, thus, consequent use of thionamides in females. The indication for thionamide was hyperthyroidism due to Graves disease in all except for one patient with multinodular goiter. The doses of methimazole used vary across reports, ranging from 10 to 30 mg, whereas carbimazole-related pancreatitis has been reported with 30 to 45 mg carbimazole. However, our patient developed pancreatitis even on a lower dose of 15 mg. The onset of pancreatitis from drug exposure is usually a few weeks, with earlier onset (hours to days) in patients who have been rechallenged. Notably, 3 of 10 patients in the literature did not have features of pancreatitis on imaging, and the diagnosis was based on clinical and biochemical parameters. The pancreatic enzymes normalized within days or weeks of cessation of exposure; likewise, in our patient, complete resolution was observed in 6 days. The management of hyperthyroidism in patients with thionamide-induced pancreatitis reported in the literature includes propylthiouracil, potassium iodide, and radioiodine ablation.

**Table 2.** Case reports of thionamide-related pancreatitis reported in the literature

Author Year	Age/sex Ethnicity	Diagnosis	Drug Dose	Interval for onset of pancreatitis	Findings of pancreatitis on imaging	Time to normalization of enzymes
Taguchi 1999	66/F Japanese	GD	MMI 30 mg	3 wk	No	Amylase 6 d Lipase 10 d
Marazuela 2002	33/F Spanish	GD	CMZ 45 mg	4 wk	Yes	—
Su and Zou 2008	19/F Chinese	GD	MMI 10 mg	10 wk	—	—
Chng 2011	70/F Asian	GD	CMZ 30 mg	2 wk	Yes	—
Yang 2012	18/F Chinese	GD	MMI 20 mg	4 d	No	—
Abraham 2012	80/F White	—	MMI 10 mg	12 wk	Yes	Lipase 4 d
Jung 2014	51/M Korean	GD	MMI 20 mg	2 wk	Yes	Amylase 17 d
Agito 2015	51/F White	MNG	MMI 10 mg	3 wk	Yes	Lipase 10 d
Kikuchi 2018	76/F Japanese	GD	MMI 10 mg	3 wk	Yes	Lipase 3 d
Yoshimura 2022	72/F Japanese	GD	MMI 15 mg	2 wk	No	Amylase 26 d Lipase 98 d
Our case	41/M Indian	GD	CMZ 15 mg	2 weeks	Yes	Amylase 6 d Lipase 6 d

Abbreviations: CMZ, carbimazole; F, female; GD, Graves disease; M, male; MMI, methimazole; MNG, multinodular goiter.

The mechanism of methimazole- and carbimazole-induced pancreatitis is likely due to an autoimmune reaction to the drugs, as evidenced by earlier onset in rechallenged patients. The underlying reason could be direct hypersensitivity to the molecule or toxic metabolites (3). The European Medicine Agency (EMA) issued a recommendation warning in January 2019 indicating a potential risk of acute pancreatitis with methimazole based on evidence from case reports (EMA/PRAC/826440/2018). In addition to data from case reports, there are a few cohort studies. A cohort study from the previously cited Taiwan database found no correlation between methimazole and carbimazole exposure (7). However, this study did not consider the duration of time before exposure to the drug. Two cohort studies from Denmark and Italy suggest a higher risk of pancreatitis with methimazole exposure, especially within 3 months of exposure (8, 9). The Italian study further demonstrated no sex difference or relation to dose, but the risk increased with age. Nonetheless, the risk is small, about 0.02% to 0.56%. The study by Yoshimura et al identified (3) HLA DRB1*08:03 in patients with methimazole-induced pancreatitis, as previously described with agranulocytosis. However, in our patient, this HLA haplotype was not observed. Based on a review of case reports, the evidence of carbimazole-induced pancreatitis is graded as Ia by the Badalov classification, updated by Wolfe et al (6). As per the Naranjo algorithm for causality, the adverse drug reaction probability scale is probable (10). The case was reported to the Pharmacovigilance Programme of India and Vigiflow portal identification number IN-IPC 300759501.

To conclude, we report a case of probable carbimazole-induced pancreatitis. Even though it is a rare occurrence, patients taking carbimazole who report abdominal discomfort and vomiting should be evaluated for pancreatitis.

## Learning Points

- Carbimazole can be associated with pancreatitis.
- A thorough evaluation of alternative etiologies is needed when drug-induced pancreatitis is suspected.
- Carbimazole- and methimazole-associated pancreatitis onset is within weeks, which resolves in days to weeks.

## Data Availability

Original data generated and analyzed during this study are included in this published article.

## Abbreviations

HLA: human leukocyte antigen
MRCP: magnetic resonance cholangiopancreatography
T3: triiodothyronine
T4: thyroxine
TSH: thyrotropin
